# Synthesis and magnetic properties of single-crystalline Na_2-__*x*_Mn_8_O_16 _nanorods

**DOI:** 10.1186/1556-276X-6-133

**Published:** 2011-02-11

**Authors:** Changyong Lan, Jiangfeng Gong, Shijiang Liu, Shaoguang Yang

**Affiliations:** 1 Nanjing National Laboratory of Microstructures and Department of Physics, Nanjing University, 22 Hankou Road, Nanjing, 210093, China; 2 Department of Physics, Hohai University, 1 Xikang Road, Nanjing, 210098, China; 3 College of Physics and Electronic Information, Luoyang Normal College, 71 Longmen Road, Luoyang, 471022, Henan, China

## Abstract

The synthesis of single-crystalline hollandite-type manganese oxides Na_2-__*x*_Mn_8_O_16 _nanorods by a simple molten salt method is reported for the first time. The nanorods were characterized by powder X-ray diffraction, scanning electron microscopy, transmission electron microscopy, and a superconducting quantum interference device magnetometer. The magnetic measurements indicated that the nanorods showed spin glass behavior and exchange bias effect at low temperatures. The low-temperature magnetic behaviors can be explained by the uncompensated spins on the surface of the nanorods.

## Background

One dimensional (1D) nanostructures including nanobelts, nanotubes, nanowires, and nanorods have attracted much attention due to their fascinating physical and chemical properties and their potential applications in nanodevices [1,2]. Manganese oxides have a wide range of applications such as catalysts [3], ion sieves [4], and battery materials [5]. Much effort has been made to prepare low dimensional manganese oxides nanomaterials with various polymorphs [6-8]. As a novel Mn^3+^-Mn^4+ ^mixed valence system, hollandite-type compounds with chemical formula A_*x*_Mn_8_O_16 _(A = K, Rb, Ba, or Pb, etc. and *x *≤ 2) have been enthusiastically pursued for their applications in fast ionic conductors, solid state electrolytes, oxidation catalysts, and stable host materials for radioactive ions from nuclear wastes [9-12]. The crystal structure of the hollandite-type material is very porous, including 1D 2 × 2 tunnels among rigid MnO_2 _framework composed of edge-shared MnO_6 _octahedra [4,10,13]. The A ions occupy in the tunnels as guest cations and they are easily replaced by other ions [13]. Due to the special crystal structure and the mixed valence properties of Mn, these compounds show interesting magnetic and electric properties [13-16]. The formation of K_x_Mn_8_O_16 _with hollandite-type structure is very easy, since the K^+ ^cation is of the ideal dimension to fit in the 2 × 2 tunnels. But the Na^+ ^cation is on the small side to stabilize the 2 × 2 tunnels, thus hollandite Na-Mn-O compound is hard to be obtained [3]. Na_2-__*x*_Mn_8_O_16 _is known to have hollandite structure with unit-cell parameters *a *= 9.91 Å, *b *= 2.86 Å, *c *= 9.62 Å and *β *= 90.93° (JCPDS No. 42-1347), and the ion tunnel of which is along *b*-axis. To the best of our knowledge little information about this compound has been reported. Here, we report the synthesis of Na_2-__*x*_Mn_8_O_16 _nanorods by a very simple molten salt method for the first time.

Exchange bias (EB) effect is observed in the materials with good ferromagnetic (FM)/antiferromagnetic (AFM) interface, such as Ni_80_Fe_20_/Ir_20_Mn_80 _system [17]. The EB effect originates from the interfacial interaction between FM and AFM materials [18]. Recently, it was reported that 1D pure phase AFM nanomaterials exhibited EB effect at low temperatures, such as Co_3_O_4 _nanorods [19], SrMn_3_O_6-δ _nanobelts [20], CuO nanowires [21]. Since there is no FM layer in those materials, the EB effect in pure 1D AFM nanomaterials is probably related to the surface layer of the nanomaterials, which is due to the changes in the atomic coordination form a layer of disordered spins (i. e. spin glass layer) [18]. As a kind of 1D magnetic nanomaterials, the Na_2-__*x*_Mn_8_O_16 _nanorods may show novel magnetic properties. Thus the magnetic properties of Na_2-__*x*_Mn_8_O_16 _nanorods are explored and we find that the as-synthesized nanorods exhibit spin glass behavior and EB effect at low temperatures.

## Results and discussion

**Figure 1:**
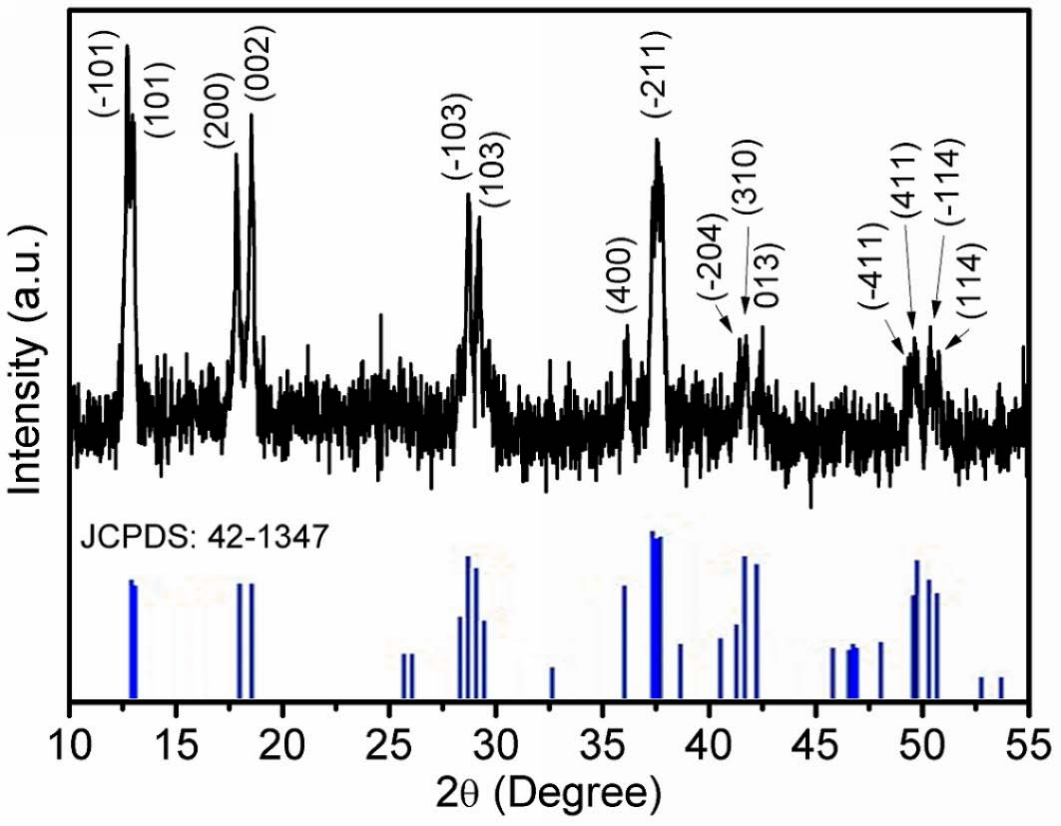
**XRD pattern of Na**_**2-x**_**Mn**_**8**_**O**_**16 **_**nanorods at room temperature**.

The X-ray diffraction (XRD) pattern of Na_2-__*x*_Mn_8_O_16 _nanorods is shown in Figure 1. The peaks can be indexed to monoclinic phase of Na_2-__*x*_Mn_8_O_16 _(JCPDS No. 42-1347). No secondary phase is observed, indicating pure phase Na_2-__*x*_Mn_8_O_16 _was obtained. As the Na^+ ^cation is on the small side to stabilize the 2 × 2 tunnels compared with K^+ ^cation, it is difficult to synthesize Na_2-__*x*_Mn_8_O_16 _[3]. In fact, we have tried to synthesize Na_2-__*x*_Mn_8_O_16 _by solid state reaction using stoichiometric amount of NaNO_3 _and MnCO_3 _as starting materials (suppose *x *= 0 in the formula Na_2-__*x*_Mn_8_O_16_), but no Na_2-__*x*_Mn_8_O_16 _phase could be obtained. In order to keep the 2 × 2 tunnel structure stable when K^+ ^cations are replaced by Na^+ ^cations, more Na^+ ^cations are needed. In the high-temperature liquid molten salt, there is a large quantity of free Na^+ ^cations. Suppose the unstable 2 × 2 tunnels formed in the molten salt first, then the Na^+ ^cations can go into the tunnels. The excess of Na^+ ^cations can guarantee there are enough Na^+ ^cations in the 2 × 2 tunnels to make the tunnels stable. Based on the above discussion, the *x *in Na_*x*_Mn_8_O_16 _should be larger than that in K_*x*_Mn_8_O_16_. The *x *in K_*x*_Mn_8_O_16 _is 1.5 [14], while the *x *in Na_*x*_Mn_8_O_16 _obtained from the EDS result discussed later in this letter is 1.74, which confirms the above conclusion.

**Figure 2:**
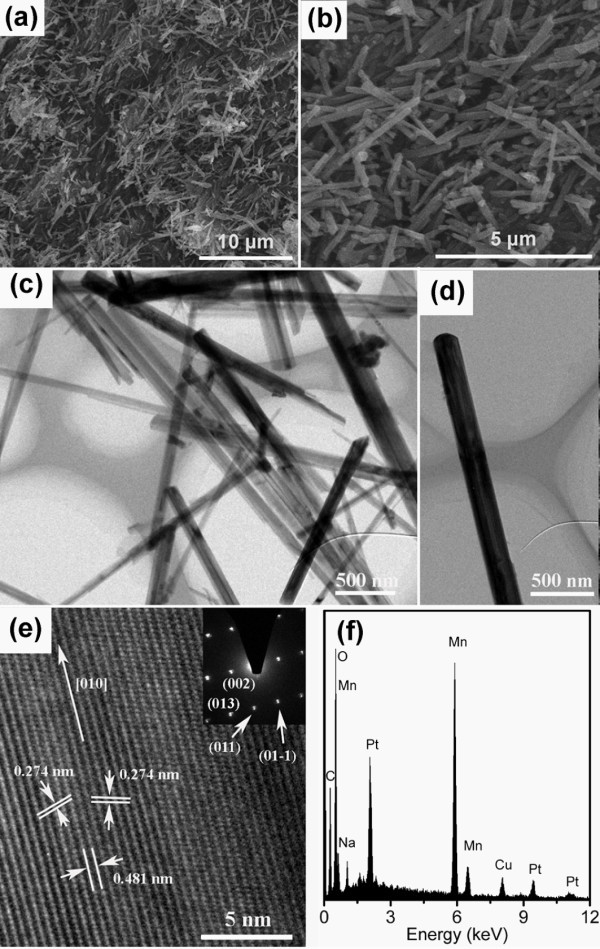
**SEM and TEM images**. **(a) **Low-magnification SEM image of Na_2-__*x*_Mn_8_O_16 _nanorods; **(b) **high-magnification SEM image of Na_2-__*x*_Mn_8_O_16 _nanorods; **(c) **TEM image of Na_2-__*x*_Mn_8_O_16 _nanorods; **(d) **TEM image of a single Na_2-__*x*_Mn_8_O_16 _nanorod; **(e) **HRTEM image of the Na_2-__*x*_Mn_8_O_16 _nanorod, the inset of **(e) **is the corresponding SAED pattern of the nanorod. **(f) **EDS spectrum of the Na_2-__*x*_Mn_8_O_16 _nanorods. C peak originates from conductive adhesive, Cu peak originates from Cu sheet, and Pt peaks originate from sputtered Pt layer. (a) scale bar 10 μm, (b) scale bar 5 μm, (c) (d) scale bar 500 nm, (e) 5 nm

A low-magnified scanning electron microscopy (SEM) image of Na_2-__*x*_Mn_8_O_16 _nanorods is shown in Figure 2a. From the SEM image, it can be found that large quantity of nanorods was obtained. The average diameter of the nanorods is about 200 nm from the high-magnified SEM image as shown in Figure 2b. The transmission electron microscopy (TEM) image shown in Figure 2c indicates that the product mainly consists of solid-rod-like structures and the average diameter of the nanorods is about 200 nm, consisting with the SEM results. The TEM image of a single nanorod is shown in Figure 2d. The high-resolution TEM (HRTEM) image taken from a part of the single nanorod is shown in Figure 2e. Clear lattice fringes in Figure 2e indicate a high crystallinity of the nanorod. The lattice spacings of 0.481 and 0.274 nm are recognized and ascribed to the (002) and (011) (or (01-1)) planes of the monoclinic phase of Na_2-__*x*_Mn_8_O_16_, respectively. The corresponding selected area electron diffraction (SAED) pattern taken from the same nanorod can be indexed to the reflections of the monoclinic phase of Na_2-x_Mn_8_O_16 _as shown in the inset of Figure 2e. The clear diffraction spots indicate the high crystallinity of the nanorod, which is consistent with HRTEM result. Combing the HRTEM and SAED results, it can be concluded that the growth direction of the nanorod is along [010], which is the tunnel direction of the compound. The composition of the as-synthesized nanorods was determined by EDS. Figure 2f shows the EDS spectroscopy. The chemical components of the nanorods are Na, Mn, and O with the ratio 7.24:33.38:59.38. The ratio of O/Mn is close to 2, which is consistent with the chemical formula. The chemical formula calculated from the EDS result is Na_1.74_Mn_8_O_16_.

**Figure 3:**
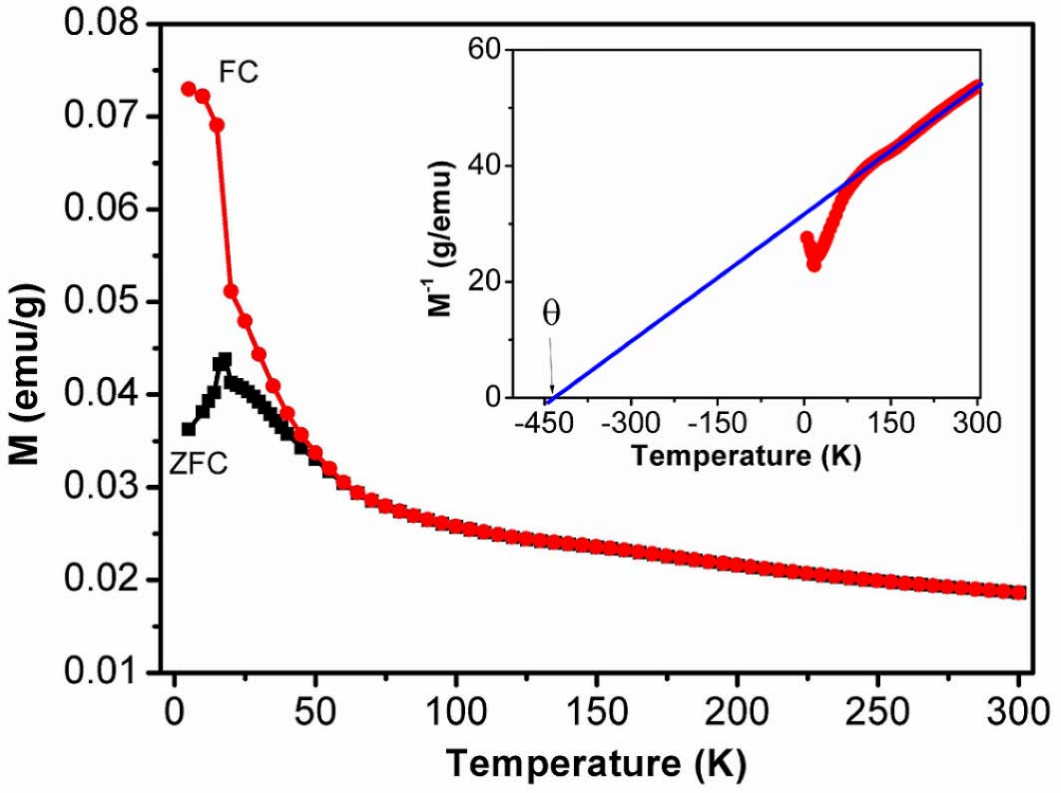
**Temperature dependence of magnetization of Na**_**2-**__***x***_**Mn**_**8**_**O**_**16 **_**nanorods for ZFC and FC measurements under a magnetic field of 500 Oe**. The inset shows the inverse magnetization versus temperature. Solid line represents linear fit between 90 and 300 K.

The magnetic properties of the Na_2-__*x*_Mn_8_O_16 _nanorods were explored. Figure 3 shows the temperature-dependent magnetization curves of the nanorods in zero-field-cooled (ZFC) and field-cooled (FC) processes with an applied magnetic field of 500 Oe. The ZFC magnetization curve shows a sharp peak near 19 K (*T*_B_) and an evident separation from the FC curve below *T*_B_, suggesting a spin-glass-like behavior at low temperatures [16,19-21]. Such behavior can be attributed to uncompensated surface spins in the 1D nanostructures [16,19-21]. The linear fit for the temperature dependence of the inverse magnetization shows that the product exhibits Curie-Weiss behavior above about 90 K and gives an extrapolated Curie-Weiss temperature (*θ*) of about -440 K as shown in the inset of Figure 3. The large negative Curie-Weiss temperature indicates the AFM interactions in Na_2-__*x*_Mn_8_O_16 _are very strong.

**Figure 4:**
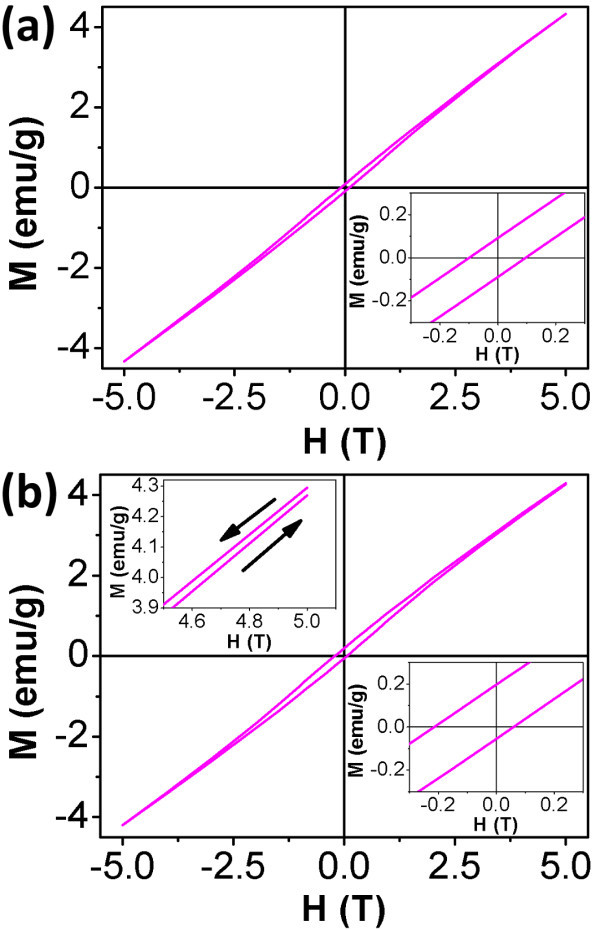
**Magnetization as a function of magnetic field at 5 K for Na_2-x_Mn_8_O_16 _nanorods**. **(a) **after ZFC process; **(b) **after FC process with an applied magnetic field of 5 T. The inset in the lower right corner of (a) and (b) shows the magnified part of the corresponding loop in the low field ranges. The inset in the upper left corner of (b) shows the high field irreversibility of magnetization on the right-hand side.

Hysteresis loops of the Na_2-__*x*_Mn_8_O_16 _nanorods recorded at 5 K under ZFC and FC conditions are shown in Figure 4a, and 4b, respectively. For the FC loop, the sample was cooled from room temperature under an applied magnetic field of 5 T. As can be seen in Figure 4a the hysteresis loop recorded under ZFC conditions is symmetrical, centers about the origin, and exhibits a coercive field of about 980 Oe. On the contrary, for the FC process an asymmetry magnetic hysteresis loop (Figure 4b) exhibiting shifts both in the field and magnetization axes as well as an enhanced coercivity (approximately 1,375 Oe) is observed, which indicates the existence of EB phenomenon. The EB effect can be explained on the basis of a phenomenological core-shell model where the core shows AFM behavior and the surrounding shell possesses a net magnetic moment due to a large number of uncompensated surface spins [19-21]. This is different from ordinary case, where a good AFM/FM interface is needed, such as Ni_80_Fe_20_/Ir_20_Mn_80 _system [17]. The shift to positive magnetization axis for the FC loop suggests the presence of a unidirectional exchange anisotropy interaction, which drives the FM domains back to the original orientation when the field is removed [20,21]. The strength of this anisotropy is measured by the EB field *H*_E _which is defined as *H*_E _= -(*H*_1 _+ *H*_2_)/2, where *H*_1 _and *H*_2 _are left and right coercive fields, respectively. The EB field for the FC process is about 770 Oe. The remanence asymmetry *M*_E _is defined as the vertical axis equivalent to *H*_E_. Thus the *M*_E _and remanent magnetization *M*_r _under the FC mode are about 0.071 and 0.126 emu/g, respectively. The enhanced coercivity for the FC loop is ascribed to the development of the exchange anisotropy. In the case of an AFM with small anisotropy, when the FM rotates it drags the AFM spins irreversibly, hence increasing the FM coercivity [18].

The spin-glass-like behavior of the surface can also be clearly observed for the opening in the upper right side of the FC hysteresis loop, which is shown in the upper left inset of Figure 4b. This indicates that we have a loss of magnetization during one hysteresis cycle. A similar phenomenon has been observed in Co_3_O_4 _nanowires [19]. This striking experimental feature is observed here because of the large amount of measured material and due to the absence of additional ferromagnetic materials which could mask the observation of the interfacial spins behavior [19]. The EB effect induced by surface effects of the nanorods suggests that Na_2-__*x*_Mn_8_O_16 _nanorods may find potential application in multifunctional spintronic devices [22].

## Conclusions

In summary, single-crystalline Na_2-x_Mn_8_O_16 _nanorods were synthesized by a simple molten salt method for the first time. SEM and TEM images show that the nanorods are about 200 nm in width and several micrometers in length. HRTEM and SAED indicate the single-crystalline of the nanorods. The growth direction of the nanorods is along the tunnel direction of the hollandite structure. The chemical formula of the nanorods can be written as Na_1.74_Mn_8_O_16 _calculated from EDS result. Magnetic measurements indicate that the nanorods show spin glass behavior and EB effect at low temperatures. The low-temperature magnetic behaviors can be explained by the uncompensated surface spins of the nanorods.

## Methods

In a typical procedure, MnCl_2_•4H_2_O and NaOH (1:2 in molar) were dissolved in distilled water, respectively. Then NaOH aqueous solution was added to MnCl_2 _aqueous solution slowly with constant magnetic stirring. The precipitation was filtered and washed several times and then dried at 90°C for 24 h. After being dried, black powder was obtained. 0.1 g of the obtained black powder was mixed with 5 g NaNO_3 _and ground for 20 min in an agate mortar by hand. The mixture was then placed in a corundum crucible and annealed at 550°C for 6 h. The product was collected after naturally cooling the furnace to room temperature and then washed several times with distilled water to remove residual NaNO_3_. The obtained black powder was dried at 90°C for 24 h.

XRD patterns were collected using a Philips X'Pert diffractometer with Cu Kα irradiation at room temperature. For the SEM characterization, the product was pasted on a Cu sheet with conductive adhesive. A thin layer of Pt was sputtered on the sample to enhance its conductivity for the facility of SEM measurements. SEM and EDS pattern were carried out in a Hitachi-S-3400N II instrument. In further characterization, TEM images, HRTEM images, and SAED were obtained in a Philips Tecnai F20 instrument, operating at 200 kV. Magnetic properties were obtained in a superconducting quantum interference device magnetometer.

## Competing interests

The authors declare that they have no competing interests.

## Authors' contributions

CYL conceived of the study, synthesized the materials, analysed the obtained data and drafted the manuscript. GJF helped in obtaining the transmission electron microscopy related images. SJL carried out the magnetic measurements. SGY participated in discussing the results and helped to draft the manuscript. All authors read and approved the final manuscript.
